# Attributable risk of hospital admissions for ischemic stroke due to ambient air pollution: a time-series study in Zhangzhou, China

**DOI:** 10.3389/fpubh.2026.1850457

**Published:** 2026-06-17

**Authors:** Yanhu Ji, Lina Zhang, Yiqi Liu, Yubin Chen, Tongjun Chen, Shengwen Wu, Liang Song

**Affiliations:** 1 Department of Clinical Medicine, Zhangzhou Health Vocational College, Zhangzhou, Fujian, China; 2 Zhangzhou Municipal Hospital of Fujian Province, Zhangzhou, Fujian, China; 3 The Third Hospital of Zhangzhou, Zhangzhou, Fujian, China

**Keywords:** air pollution, attributable burden, hospital admissions, ischemic stroke, time series study

## Abstract

**Background:**

This study aimed to examine the association between ambient air pollution and hospital admissions for ischemic stroke, as well as the attributable disease burden, in Zhangzhou, a subtropical city in China.

**Methods:**

Daily data on hospital admissions for ischemic stroke, meteorological factors, and ambient air pollutants (PM_2_.5, PM10, SO_2_, NO_2_, CO, and O3) were collected in Zhangzhou from 2020 to 2023. A Poisson generalized additive model (GAM) was used to evaluate the acute effects of air pollutants on ischemic stroke admissions, with stratification by sex, age, and season. Attributable fractions (AFs) and attributable numbers (ANs) were further estimated based on the World Health Organization (WHO) Air Quality Guidelines and Chinese air quality standards.

**Results:**

Ischemic stroke admissions were significantly associated with exposure to PM_2.5_, PM_10_, NO_2_ and O_3_, but not with SO_2_ or CO. For per 10 μg/m3 increment in PM_2.5_, PM_10_, NO_2_, and O_3_ at lag01, the corresponding relative risks (RRs) and 95% confidence intervals (CIs) were 1.0356 (1.0107–1.0611), 1.0196 (1.0052–1.0341), 1.0387 (1.0045–1.0740), and 1.0106 (1.0030–1.0183), respectively. These associations were stronger in males, individuals under 65 years of age, and during the cold season. Overall, 7.0% (854 cases) of ischemic stroke admissions in Zhangzhou were attributable to air pollutant concentrations exceeding the WHO Air Quality Guidelines.

**Conclusions:**

Short-term exposure to PM_2.5_, PM_10_, NO_2_, and O_3_ was significantly associated with increased hospital admissions for ischemic stroke and contributed to a certain disease burden in Zhangzhou. Our findings highlight the need for effective air pollution control strategies to reduce the related health burden.

## Introduction

Ischemic stroke is an acute cerebrovascular disease characterized by local brain tissue necrosis resulting from ischemia and hypoxia due to impaired cerebral blood circulation, and it represents the most common subtype of stroke (1, 2). It features high incidence, high disability rate, and high mortality, thus imposing a severe threat to public health (3, 4). Since the beginning of the 21st century, the global incidence of ischemic stroke has exhibited a divergent trend: a gradual decline in high-income countries and a substantial increase in low- and middle-income countries (5). Data from the 2019 Global Burden of Disease (GBD) study showed that there were more than 7.63 million incident cases of ischemic stroke worldwide, with 77.19 million prevalent cases and 3.29 million deaths (3). In China, ischemic stroke has become the leading cause of premature death among the general population, with an estimated annual increase of 10% in incidence (6). In 2019, China reported 2.87 million new ischemic stroke cases, 1.07 million deaths, and 21.39 million disability-adjusted life years (DALYs) (7), which not only substantially increased the public health burden but also imposed a considerable economic burden on patients' families and society.

Air pollution is a ubiquitous environmental risk factor with certain public health impacts. Air pollutants exert diverse adverse effects on human health, and existing evidence indicates that they primarily damage the respiratory (8), cardiovascular (9) and nervous systems (10). Statistically, approximately 4.2 million premature deaths worldwide occur annually due to exposure to ambient air pollution (11). Scholars have conducted extensive studies on the association between air pollution and ischemic stroke. For example, Yazdi et al. reported that long-term exposure to air pollutants significantly increases the risk of stroke among older American adults (12). A study conducted in New England found that each 10 μg/m3 increment in the average PM_2.5_ concentration on the day of onset and the preceding 5 days was associated with a 2.6% increase in stroke incidence (95% CI: 0.4%−4.7%) (13). Another Danish cohort study demonstrated that each interquartile range (IQR) increase in long-term NO_2_ exposure was associated with a 1.05-fold higher risk of stroke events (95% CI: 0.99–1.11) and a 1.22-fold higher risk of 30-day post-stroke mortality (95% CI: 1.00–1.50) (14). In addition, a meta-analysis noted that each 5 μg/m3 increase in PM_2.5_ concentration was related to a 6% higher risk of stroke incidence and a 12.5% higher risk of stroke mortality (15). Nevertheless, several studies have reported inconsistent findings. For instance, a nationwide study in South Korea observed no significant association between PM_10_ and ischemic stroke, with the exception of the cardiogenic subtype (16). A Spanish study also failed to confirm a significant relationship between PM_2.5_ and ischemic stroke (17). Similarly, studies conducted in Canada (18), the United Kingdom (19), the United States (20), and Tianjin, China (21) did not identify significant adverse effects of air pollutants on ischemic stroke. At present in China, although there is some evidence investigating the association between air pollution and ischemic stroke, most existing studies have focused on industrial cities in northern China or first-tier cities (21–23). There is a lack of research on the disease burden of ischemic stroke attributed to air pollutants, as well as a shortage of relevant studies in small and medium-sized low-pollution coastal cities in southern Fujian.

As a coastal city in the subtropical monsoon region, Zhangzhou has unique characteristics in terms of air pollution, climate and population structure. This study aims to supplement research evidence under the low-pollution background in southeastern China, clarify the region-specific association, and provide a scientific basis for the precise prevention and control of ischemic stroke in this area. This study has the following three research objectives: (a) To examine the associations between six common air pollutants and hospital admissions for ischemic stroke; (b) To identify potential susceptible populations through stratification by sex, age, and season; (c) To estimate the disease burden of ischemic stroke attributable to air pollutant concentrations exceeding the World Health Organization (WHO) Air Quality Guidelines and Chinese national air quality standards.

## Materials and methods

### Study area

Zhangzhou is situated in southern Fujian Province, China, with jurisdiction over four municipal districts (Xiangcheng, Longwen, Longhai, and Changtai), seven counties (Zhangpu, Yunxiao, Zhao'an, Dongshan, Nanjing, Pinghe, and Hua'an) and five development zones (Zhangzhou Development Zone, Changshan Development Zone, Gulei Development Zone, Taiwanese Investment Zone, and Zhangzhou High-tech Zone). The city covers a land area of 12,600 square kilometers and a marine area of 18,600 square kilometers, with a coastline of 715 kilometers and 143 islands along the coast. In 2024, Zhangzhou had a permanent population of approximately 5.063 million and a regional gross domestic product (GDP) of 606.371 billion yuan. Located near the Tropic of Cancer, Zhangzhou features a subtropical monsoon humid climate with warm temperatures, abundant rainfall, mild winters, and moderate summers.

## Data collection

Daily hospital admissions for ischemic stroke from 1 January 2020 to 31 December 2023 were extracted from the medical record information systems of two large tertiary hospitals in Zhangzhou, namely Zhangzhou Municipal Hospital of Fujian Province and the Third Hospital of Zhangzhou. These two hospitals represent the highest-tier medical institutions in Zhangzhou, with standardized diagnostic and treatment protocols. Specifically, the Third Hospital of Zhangzhou has 1,700 inpatient beds; Zhangzhou Municipal Hospital consists of four branches (the Headquarters Campus, Xiangcheng Campus, Longwen Campus, and Chaoyang Branch), with a total construction area of more than 530,000 square meters and 2,000 available inpatient beds. All ischemic stroke cases were double-checked and confirmed by experienced clinicians and professional technical staff. We extracted the following information for each patient: admission date, sex, age, and the corresponding International Classification of Diseases, 10th Revision (ICD-10) codes. The daily number of ischemic stroke admissions during the study period was defined based on the following inclusion and exclusion criteria: (a) only patients with a permanent residence in Zhangzhou were included, and cases with non-Zhangzhou residence or incomplete clinical data were excluded; (b) diagnoses were strictly in accordance with the ICD-10 code I63 for ischemic stroke; (c) only first admission cases were included, and readmission cases were excluded.

Meteorological data for the study period, including daily average temperature (°C) and daily average relative humidity (%), were retrieved from the Chinese Meteorological Science Data Sharing Service Network (http://www.escience.gov.cn). Daily air pollutant concentration data were obtained from the China Air Quality Real-time Monitoring Platform (https://www.aqistudy.cn/), including the 24-h average concentrations of PM_2.5_, PM_10_, SO_2_, NO_2_, CO, as well as the maximum 8-hour average concentration of O_3_. These air pollution data were obtained from certified, uniformly distributed national monitoring stations, ensuring spatial representativeness, and standard methods and regular quality control were adopted to minimize measurement bias.

### Statistical analysis

In this study, we first evaluated the associations between various air pollutants and ischemic stroke admissions, followed by subgroup analyses stratified by sex, age, and season. Additionally, we estimated the disease burden attributable to exposure to air pollutant concentrations exceeding the WHO Air Quality Guidelines and China's primary air quality standards.

The daily number of ischemic stroke admissions generally followed an overdispersed Poisson distribution; therefore, a Generalized Additive Model (GAM) with a quasi-Poisson link function was used to investigate the associations between air pollutants and ischemic stroke admissions (24). Referring to previously published studies, the model parameters were set as follows: (a) Long-term and seasonal trends were controlled using natural spline (ns) functions with 7 degrees of freedom (df) per year (25, 26); (b) Potential confounding effects of daily mean temperature and relative humidity were adjusted for using natural cubic spline functions with 3 df per year (27, 28); (c) The model also adjusted for day-of-week (DOW) effects and holiday effects. The model constructed in this study is presented below (Equation 1):


$$\begin{array}{l} LogE\left(Yt\right)\;=\beta Pt+ns\;\left(Temp,3\right)+ns\left(RH,3\right)+\;Holiday\\ \;+DOW+ns\left(time,7\times 4\right)+\mathrm{int}ercept \end{array}$$ (1)

In the above model, Temp and RH represent the daily average ambient temperature and relative humidity, respectively. *t* denotes the observation day, E(Y*t*) is the expected number of daily ischemic stroke admissions on day *t*, P_t_ is the concentration of the air pollutant, and β is the log-relative rate of the exposure-response relationship between air pollutants and ischemic stroke.

To test the robustness of the model, we applied the following strategies. First, single-day lag structures (lag 0 to lag 5) and multiple-day moving average lag structures (lag01 to lag05) were used to examine the effects of air pollution on daily ischemic stroke admissions (29). For instance, lag 0 indicates exposure on the current day of admission, whereas lag01 represents the moving average exposure on the current and previous days. Second, sensitivity analyses were performed by changing the degrees of freedom for time trends (6–9 *df* per year) and for mean temperature and relative humidity (3–5 *df* per year). Third, two-pollutant models were constructed after adjusting for other co-pollutants.

In addition, subgroup analyses were conducted by sex (male and female), age (< 65 years and ≥65 years), and season (warm season and cold season). Between-group differences were tested using the following formula (Equation 2) (30):


$$\begin{array}{l} \left(Q1+Q2\right)\;\pm \;1.96\sqrt{SE_{1}^{2}+SE_{2}^{2}}. \end{array}$$ (2)

Taking sex groups as an example, if Q_1_ and SE_1_ represent the effect estimate and standard error for males, respectively, then Q_2_ and SE_2_ represent the corresponding values for females.

Finally, we estimated the disease burden of ischemic stroke admissions attributable to concentrations of air pollutants exceeding the WHO Air Quality Guidelines and Chinese national air quality standards. In single-pollutant models, the maximum exposure-response coefficient for ischaemic stroke admissions was used to calculate the attributable fraction (AF) and number (AN). The formula is shown below (Equation 3) (31):


$$\begin{array}{l} ANt\;=\;Nt\;\times \;\left[1\;-\;exp\;\left(-\;\beta \right)\;\times \;\left(Pt\;-\;Standard\right)\right] \end{array}$$ (3)

In Equation 3, *t* denotes day *t*; *N*_*t*_ is the number of ischaemic stroke admissions on day *t*; β is the exposure-response coefficient derived from Equation 1; *P*_*t*_ *- Standard* is the concentration of air pollutant on day *t* minus the reference concentration; and *AN*_*t*_is the number of ischaemic stroke admissions on day *t* attributable to concentrations exceeding the reference level.

All statistical analyses in this study were performed using R software (version 4.0.2), and the generalized additive models were fitted using the “mgcv” package. *P* value < 0.05 was considered statistically significant. Results are presented as relative risks (RR) and 95 % confidence intervals (CI) for daily ischemic stroke admissions associated with a 10 μg/m3 increment in air pollutant concentrations (or 1 mg/m3 for CO).

## Results

Descriptive statistics of daily ischemic stroke admissions, meteorological factors, and air pollutant variables from 2020 to 2023 are presented in Table 1. A total of 12,255 ischemic stroke admissions were identified. Of these, 63.85% (7,825 cases) were male and 36.15% (4,430 cases) were female. With regard to age, 38.71% of cases (4,743 cases) were in patients under 65 years, while 61.29% (7,512 cases) were aged 65 years and older. The number of hospital admissions in the warm season was slightly higher than that in the cold season (6,464 vs. 5,791). During the study period, the daily average concentrations of PM_2.5_, PM_10_, NO_2_, SO_2_, and CO, as well as the maximum 8-h average concentration of O3, were 22.92 μg/m3, 43.75 μg/m3, 22.25 μg/m3, 6.53 μg/m3, 0.55 mg/m3, and 93.96 μg/m3, respectively. The corresponding daily average temperature and relative humidity were 22.83 °C and 74.61%, respectively.

**Table 1:** Basic description of daily ischemic stroke hospitalizations, meteorological variables and air pollutants in Zhangzhou, 2020–2023.

Variables	Sum	Mean	SD	Minimum	P10	P25	P50	P75	P90	Maximum
Ischemic stroke	12,255	8.39	3.15	0	5	6	8	10	12	20
Males	7,825	5.35	2.42	0	2	4	5	7	9	15
Females	4,430	3.03	1.81	0	1	2	3	4	5	10
< 65 years old	4,743	3.25	1.84	0	1	2	3	4	6	11
≥65 years old	7,512	5.14	2.38	0	2	3	5	7	8	15
Warm season	6,464	4.42	4.89	0	0	0	2	9	11	20
Cold season	5,791	3.96	4.59	0	0	0	0	8	11	20
Air pollutants										
PM_2.5_ (μg/m^3^)	–	22.92	11.76	4	9	13	21	30	40	67
PM_10_ (μg/m^3^)	–	43.75	20.52	5	20	28	40	58	73	119
NO_2_ (μg/m^3^)	–	22.25	10.02	3	10	15	20	29	36	58
SO_2_ (μg/m^3^)	–	6.53	2.07	2	4	5	6	8	9	16
O_3_ (μg/m^3^)	–	93.96	34.60	0	50	69	93	119	140	193
CO (mg/m^3^)	–	0.55	0.15	0.2	0.4	0.4	0.5	0.6	0.8	1.2
Meteorological variables										
Mean temperature (°C)	–	22.83	5.97	7.4	14.8	17.7	23.6	28.3	30.1	33.4
Relative humidity (%)	–	74.61	11.59	31.0	60.0	67.0	75.0	82.0	91.0	99.0

PM_2.5_, particulate matter with aerodynamic diameter less than 2.5 μm; PM_10_, particulate matter with aerodynamic diameter less than 10 μm; SO_2_, Sulfur dioxide; NO_2_, nitrogen dioxide; CO, Carbon monoxide; O_3_, Ozone. P_10_, P_25_, P_50_, P_75_, P_90:_ the10th, 25th, 50th, 75th, 90th percentile; *SD*, standard deviation; Cold season, November to April of the next year; Warm season, May to October.

Supplementary Table A1 presents the Spearman correlation coefficients between meteorological factors and air pollutants in Zhangzhou, China. A strong positive correlation was observed between PM_2.5_ and PM_10_ (*r*_*s*_ = 0.90, *P* < 0.001). Weak correlations were found between CO and SO_2_, O3 and NO_2_, and O3 and CO, while the other air pollutants were moderately positively correlated with each other. Daily average temperature was positively correlated with SO_2_ (*r*_*s*_ = 0.017) and O3 (*r*_*s*_ = 0.19, *P* < 0.001) but inversely correlated with the other pollutants. Relative humidity was positively correlated with CO (*r*_*s*_ = 0.19, *P* < 0.001) and negatively correlated with the other pollutants.

The time-series distributions of daily ischemic stroke admissions and environmental variables in Zhangzhou are presented in Supplementary Figure S1. It can be observed that the concentrations of all air pollutants except O3 were higher in winter than in other seasons. Furthermore, the daily number of hospital admissions for ischemic stroke exhibited an increasing trend year by year.

Table 2 displays the RRs and 95% CIs of daily ischemic stroke admissions associated with per 10 μg/m^3^ (or 1 mg/m^3^ in CO) increase in air pollutants in single-pollutant models. We found that short-term exposure to PM_2.5_, PM_10_, NO_2_ and O_3_ was significantly associated with an increased risk of daily hospitalizations for ischemic stroke. For every 10 μg/m3 increase in pollutant concentrations, the significant single-day adverse effect of PM_2.5_ lasts from lag0 (RR = 1.0307, 95% CI: 1.0091–1.0528) to lag1 (RR=1.0232, 95% CI: 1.0016–1.0452), with multi-day moving average lag days persisting from lag01 (RR=1.0356, 95%CI: 1.0107–1.0611) to lag02 (RR=1.0315, 95% CI: 1.0039–1.0599). For PM_10_, the significant single-day adverse effect occurred on the day of exposure (RR=1.0198, 95% CI: 1.0069–1.0329), with multi–day moving average lag days persisting from lag01 (RR=1.0196, 95% CI: 1.0052–1.0341) to lag02 (RR=1.0158, 95% CI: 1.0003–1.0317). For NO_2_, the significant single-day adverse effect occurred on the day of exposure (RR=1.0356, 95% CI: 1.0058–1.0663), with multi-day moving average lag days persisting from lag01 (RR=1.0387, 95% CI: 1.0045–1.0740) to lag02 (RR=1.0374, 95% CI: 1.0003–1.0758). The significant adverse effects of O_3_ occurred only at lag0 (RR=1.0114, 95% CI: 1.0044–1.0184) and lag01 (RR=1.0106, 95% CI: 1.0030–1.0183). However, no significant association was found between SO_2_ or CO and hospitalizations for ischemic stroke.

**Table 2:** Relative risks of daily ischemic stroke hospitalizations associated with per 10 μg/m^3^ (or 1 mg/m^3^ in CO) increase in air pollutants in single-pollutant models.

Lag	PM_2.5_	PM_10_	SO_2_	NO_2_	CO	O_3_
0	1.0307 (1.0091–1.0528)^*^	1.0198 (1.0069–1.0329)^*^	1.0612 (0.9447–1.1922)	1.0356 (1.0058–1.0663)^*^	1.1011 (0.9359–1.2954)	1.0114 (1.0044–1.0184)^*^
1	1.0232 (1.0016–1.0452)^*^	1.0111 (0.9986–1.0238)	1.0509 (0.9377–1.1779)	1.0238 (0.9941–1.0544)	1.0031(0.8558–1.1757)	1.0052 (0.9987–1.0117)
2	1.0040 (0.9830–1.0255)	1.0008 (0.9885–1.0132)	1.0288 (0.9184–1.1525)	1.0129 (0.9840–1.0427)	0.9573 (0.8194–1.1183)	0.9994 (0.9932–1.0057)
3	0.9843 (0.9637–1.0054)	0.9952 (0.9830–1.0075)	1.0108 (0.9026–1.1319)	1.0058 (0.9776–1.0348)	0.9054 (0.7754–1.0571)	0.9989 (0.9927–1.0051)
4	0.9909 (0.9701–1.0122)	0.9935 (0.9813–1.0058)	0.9887 (0.8838–1.1061)	0.9753 (0.9481–1.0033)	0.9278 (0.7953–1.0823)	0.9976 (0.9914–1.0038)
5	0.9827 (0.9619–1.0038)	0.9930 (0.9808–1.0054)	1.0274 (0.9193–1.1483)	0.9775 (0.9502–1.0056)	09513 (0.8157–1.1094)	0.9958 (0.9897–1.0020)
01	1.0356 (1.0107–1.0611)^*^	1.0196 (1.0052–1.0341)^*^	1.0746 (0.9417–1.2263)	1.0387 (1.0045–1.0740)^*^	1.0614 (0.8881–1.2683)	1.0106 (1.0030–1.0183)^*^
02	1.0315 (1.0039–1.0599)^*^	1.0158 (1.0003–1.0317)^*^	1.0736 (0.9305–1.2388)	1.0374 (1.0003–1.0758)^*^	1.0232 (0.8466–1.2367)	1.0078 (0.9996–1.0161)
03	1.0197 (0.9898–1.0504)	1.0113 (0.9946–1.0283)	1.0666 (0.9155–1.2427)	1.0340 (0.9949–1.0746)	0.9763 (0.7994–1.1923)	1.0061 (0.9974–1.0150)
04	1.0142 (0.9821–1.0473)	1.0075 (0.9897–1.0256)	1.0538 (0.8972–1.2378)	1.0194 (0.9790–1.0613)	0.9530 (0.7730–1.1748)	1.0045 (0.9952–1.0138)
05	1.0068 (0.9726–1.0421)	1.0046 (0.9857–1.0237)	1.0579 (0.8943–1.2514)	1.0082 (0.9667–1.0515)	0.9466 (0.7617–1.1764)	1.0025 (0.9928–1.0124)

^*^P < 0.05.

**Figure 1:**
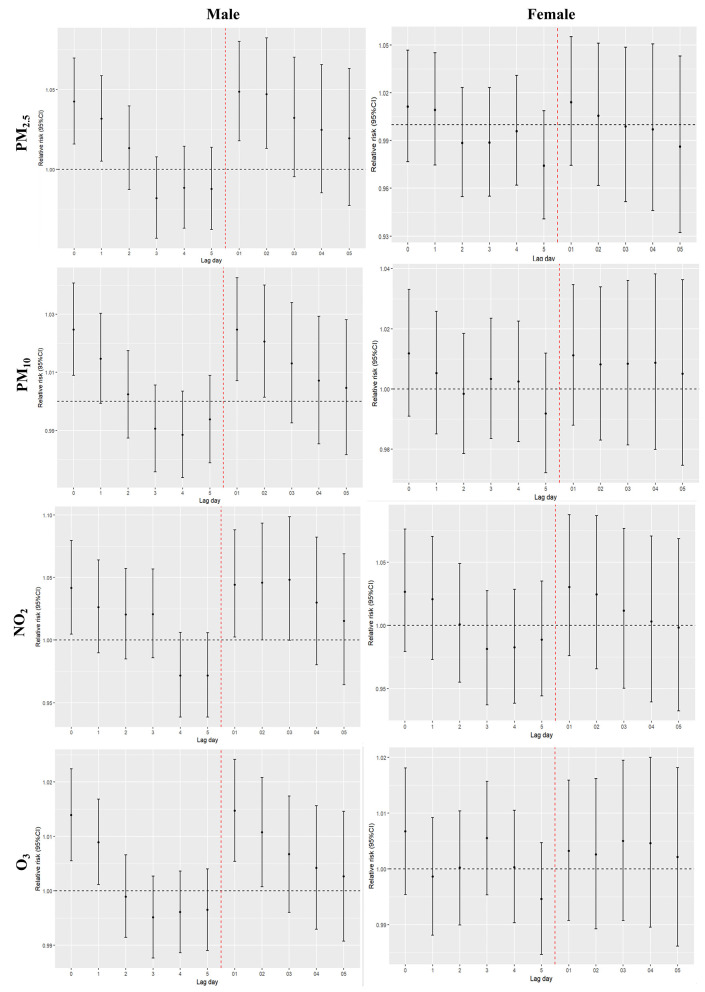
Sex-specific analyses for daily ischemic stroke admissions associated with 10 μg/m^3^ increase in pollutant concentrations.

**Figure 2:**
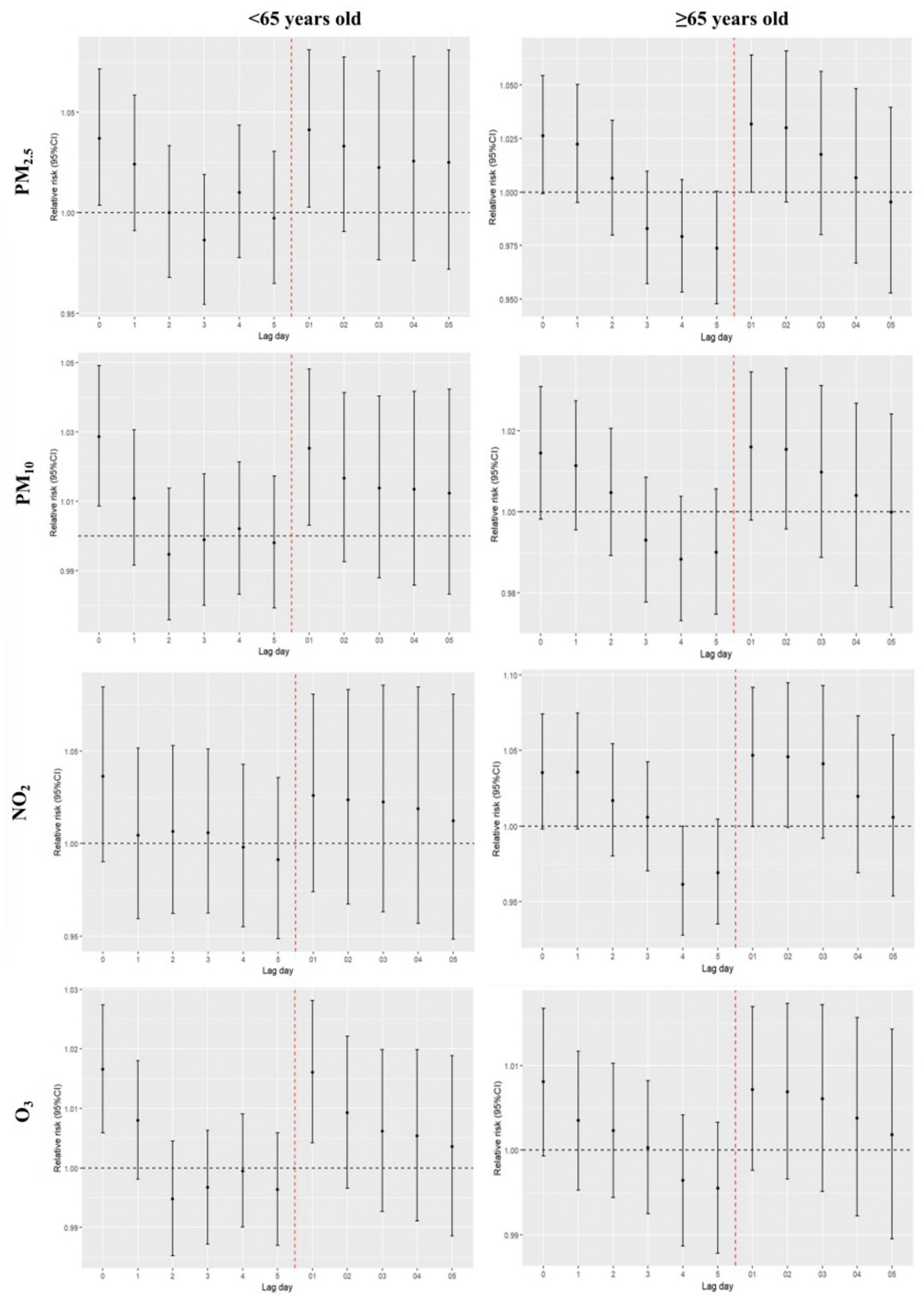
Age-specific analyses for daily ischemic stroke admissions associated with 10 μg/m^3^ increase in pollutant concentrations.

Figures 1, 2 show the results of sex- and age-specific analyses of daily ischemic stroke admissions associated with a 10 μg/m^3^ increase in air pollutant concentrations. We found that males and patients under 65 years old seem to be more susceptible to air pollutants than their corresponding groups (Tables A2–A5). In the male group, for every 10 μg/m^3^ increase in PM_2.5_, PM_10_, NO_2_ and O_3_ concentrations at lag0, the associated RRs were 1.0424 (1.0158–1.0697)?1.0247 (1.0089–1.0408)?1.0416 (1.0049–1.0797) and 1.0139 (1.0055–1.0224), respectively. In the younger group (< 65 years old), for every 10 μg/m^3^ increase in PM_2.5_, PM_10_ and O_3_ concentrations at lag01, the associated RRs were 1.0413 (1.0029–1.0812), 1.0253 (1.0031–1.0481) and 1.0161 (1.0042–1.0281), respectively.

**Figure 3:**
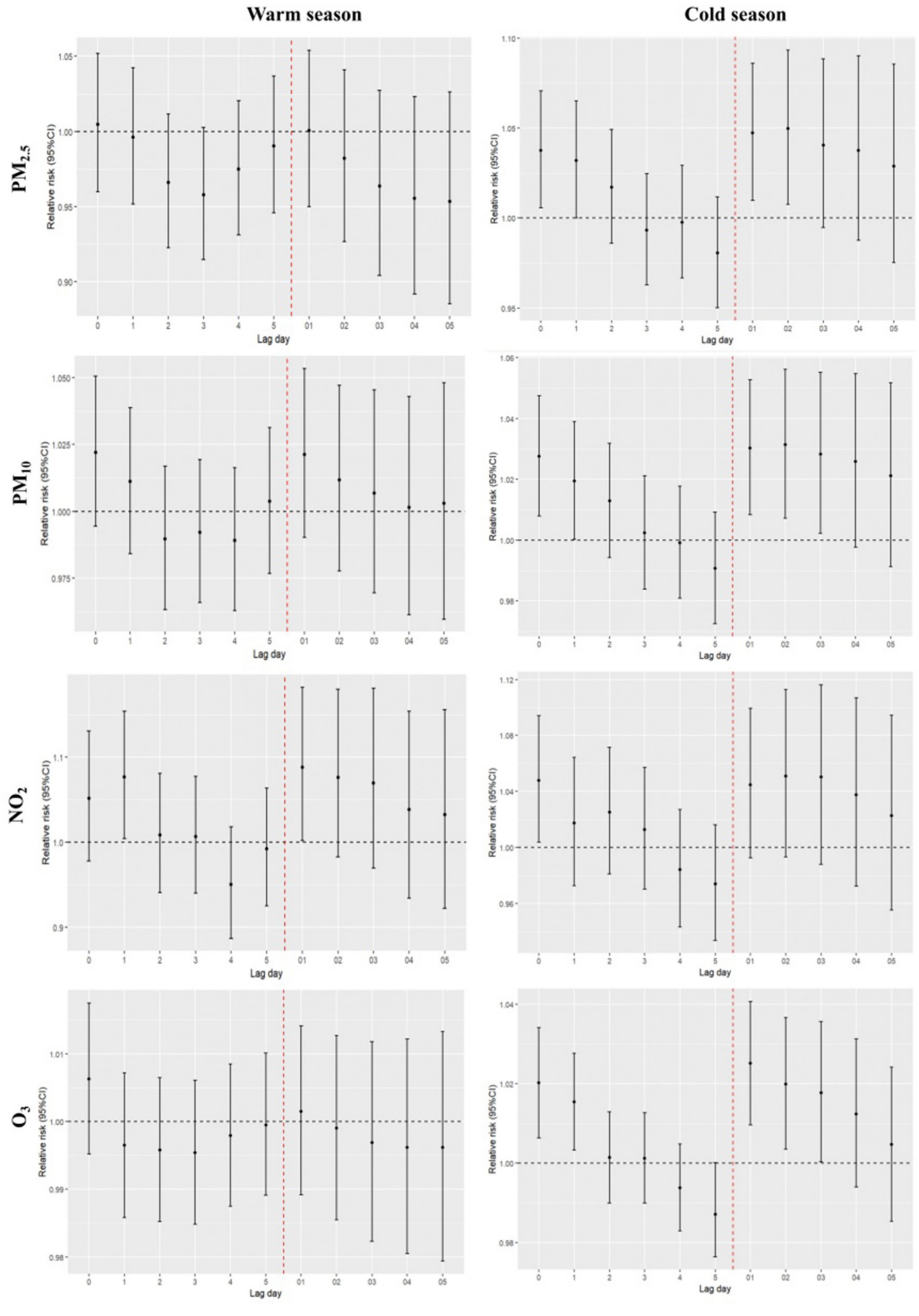
Season-specific analyses for daily ischemic stroke admissions associated with 10 μg/m^3^ increase in pollutant concentrations.

The results of the seasonal-specific analysis are shown in Figure 3. We found that the significant adverse effects of air pollutants were stronger in the cold season than in the warm season (Tables A6–A7). In the cold season group, for every 10 μg/m^3^ increase in PM_2.5_, PM_10_, NO_2_ and O_3_ concentrations at lag0, the corresponding RRs were 1.0377 (1.0058–1.0706), 1.0275 (1.0079–1.0475)?1.0479 (1.0037–1.0942) and 1.0202 (1.0063–1.0341), respectively. However, no significant adverse effects of air pollutants were observed during the warm season.

The attributable fraction (AF) and number (AN) of daily ischemic stroke hospitalizations due to air pollutants exposure by using different air quality standards are displayed in Table 3. Using WHO air quality standards as a reference, a total of 7.0% (854 cases) of daily ischemic stroke admissions were due to excess exposure to air pollutants. Specifically, 3.1% (380 cases) are due to PM_2.5_, 1.5% (182 cases) are due to PM_10_, 1.1% (131 cases) are due to NO_2_ and 1.3% (161 cases) are due to O_3_.

**Table 3:** Attributable number (AN) and fraction (AF) of daily ischemic stroke hospitalizations due to air pollutants exposure by using different air quality standards.

Air pollutants	WHO air quality guideline		China air quality primary standard	
	AN (no., 95% CI)	AF (%, 95% CI)	AN (no., 95% CI)	AF (%, 95% CI)
PM_2.5_	380 (248, 507)	3.1 (2.0, 4.1)	55 (36, 73)	0.4 (0.3, 0.6)
PM_10_	182 (123, 241)	1.5 (1.0, 1.9)	138 (93, 181)	1.1 (0.8, 1.5)
NO_2_	131 (73, 188)	1.1 (0.6, 1.5)	0 (0, 0)	0 (0, 0)
O_3_	161 (112, 210)	1.3 (0.9, 1.7)	161 (112, 210)	1.3 (0.9, 1.7)
Total	854 (556, 1,146)	7.0 (4.5, 9.4)	354 (241, 464)	2.9 (1.9, 3.8)

The strongest effects of air pollutants in single-pollutant models were used (PM_2.5_, lag01 day; NO_2_, lag01 day; PM_10_, lag0 day; O_3_, lag0 day; SO_2_, lag7 day). WHO air quality guidelines: 15 μg/m^3^ for PM_2.5_, 45 μg/m^3^ for PM_10_, 25 μg/m^3^ for NO_2_ and 100 μg/m^3^ for O_3_. China's primary standards: 35 μg/m^3^ for PM_2.5_, 50 μg/m^3^ for PM_10_, 80 μg/m^3^ for NO_2_ and 100 μg/m^3^ for O_3_. During the study period, the daily maximum concentrations of CO (1.2 mg/m^3^) and SO_2_ (16 μg/m^3^) did not exceed the WHO guidelines or China's air quality standards, so the attributable fractions of CO and SO_2_ were not calculated.

Table A8 shows the relative risk of daily ischemic stroke hospitalizations associated with per 10 μg/m^3^ increase in air pollutants in two-pollutant models. In the PM_2.5_ and PM_10_ single-pollutant models, the significant differences remained after adding other pollutants (excluding O_3_). In the O_3_ pollutant model, no significant changes in the effect values were found after adding other pollutants. However, in the NO_2_ single-pollutant model, the effect values decreased after adding other pollutants (excluding SO_2_). In addition, after adjusting the *dfs* for time trend (6–9) or for daily average temperature and relative humidity (3–5), the effects did not change significantly (Tables A9-A10).

## Discussion

Currently, the association between air pollution and the risk of ischemic stroke has attracted growing attention. However, evidence from low- and middle-income cities remains limited, particularly in subtropical regions of southeastern China. Therefore, we conducted a time-series study using GAMs to evaluate the short-term effects of ambient air pollutants on ischemic stroke hospitalizations in Zhangzhou and further estimated the corresponding attributable disease burden. The study results indicate that short-term exposure to PM_2.5_, PM_10_, NO_2_, and O_3_ is significantly associated with an increased risk of ischemic stroke admissions. These effects were notably stronger in males, individuals under 65 years of age, and during the cold season compared with their respective comparison groups. Sensitivity analyses confirmed the robustness of the associations observed between air pollutants and ischemic stroke. Using the WHO Air Quality Guidelines as the reference standard, 7.0% (854 cases) of ischemic stroke admissions were attributable to excessive exposure to air pollutants.

Our findings indicate that short-term exposure to PM_2.5_, PM_10_, NO_2_, and O_3_ is significantly associated with ischemic stroke hospital admissions in Zhangzhou. To date, no consistent conclusions have been reached regarding the specific impacts of particulate matter exposure on ischemic stroke. Existing studies have generally confirmed that both short-term particulate matter exposure (within 1 month) and long-term particulate matter exposure (for more than 3 months to several years) increase the risk of ischemic stroke (32). A nationwide study in Japan, which included 330,000 hospitalized cases, found that same-day PM_2_.5 exposure was associated with an increased risk of ischemic stroke hospitalization (33). A nationwide study in China also arrived at the same conclusion (34). In studies focusing on long-term exposure, Mahdieh et al. integrated data from U.S. medical insurance records and found that an increase in the annual average PM_2.5_ concentration was associated with an elevated risk of ischemic stroke visits (12). Another study by Huang et al. also demonstrated that increased long-term PM_2_.5 exposure concentrations significantly raised the incidence of ischemic stroke (35). In contrast, several studies have failed to identify significant adverse effects of particulate matter on ischemic stroke. A recent meta-analysis involving 16 high-quality cohort studies showed that increased PM_2.5_ concentrations were significantly associated with stroke in North America and Europe, whereas the pooled analysis results in Asia revealed no statistically significant association between the two (36). A nationwide study in South Korea indicated that PM_10_ was a risk factor for cardiogenic ischemic stroke but had no significant association with atherosclerotic or other subtypes of ischemic stroke (16). Additionally, a study in Spain found that black carbon was associated with atherosclerotic ischemic stroke but failed to identify a clear association between PM_2_.5 and ischemic stroke (17). An analysis by the O'Donnell team in Canada suggested a negative correlation between particulate matter exposure and the risk of ischemic stroke.

Most studies suggest that exposure to gaseous pollutants is positively associated with the risk of ischemic stroke. In 2014, a meta-analysis by Yang et al. based on 34 studies reported that all gaseous air pollutants except O3 were positively associated with hospitalization and mortality due to ischemic stroke (37). Several recent epidemiological studies conducted in China also support this conclusion. For example, a study in Jiangsu indicated that four gaseous air pollutants were all associated with an increased risk of mortality from ischemic stroke (38). Studies performed in Chongqing (22) and Shenzhen (23) similarly confirmed the adverse effects of gaseous pollutants on ischemic stroke risk. In addition, a study in Singapore found that elevated concentrations of CO and O3 may increase the risk of acute ischemic stroke among patients with atrial fibrillation, with this association being more pronounced in individuals aged 65 years and older and in non-smokers (39). However, findings from different regions are not entirely consistent, and some studies have not detected a significant association between gaseous pollutants and ischemic stroke. An analysis by a research team from the University of Sheffield of first-ever stroke events in South London showed that only NO_2_ was potentially associated with ischemic stroke caused by cerebral small-vessel disease, whereas no association was observed between other pollutants and other stroke subtypes (19). A large prospective cohort study of women in the United States similarly found no association between gaseous pollutants and the risk of incident ischemic stroke (20). A population-based study in Tianjin, China, reached a comparable conclusion, reporting that gaseous pollutants did not significantly increase the risk of hospital admission for ischemic stroke (21). Overall, the existing evidence regarding the association between air pollution and ischemic stroke remains limited and inconsistent, especially in developing countries. Against this background, we conducted the present study to explore the relationship between air pollution and ischemic stroke hospital admissions. The findings of this study may provide a scientific reference for government authorities to implement targeted strategies for the prevention of ischemic stroke.

This study found that males and individuals under 65 years of age are more susceptible to the adverse effects of air pollutants. Previous studies have shown that risk factors for ischemic stroke are generally more prevalent in males (40). A Danish epidemiological study indicated that cardiovascular and cerebrovascular disease-related risk factors are increasingly common among young people, resulting in a younger age of onset for ischemic stroke (41). Several potential factors may explain why males and those under 65 years are more vulnerable to air pollutants.

Males are more susceptible to air pollution due to higher levels of outdoor and occupational exposure, greater inhalation doses, and more prevalent unhealthy lifestyles. Additionally, the absence of estrogen-related vascular protective effects further increases their risk of ischemic stroke (42, 43). For individuals younger than 65 years, longer durations of outdoor activities and higher exposure levels contribute to a stronger association between air pollution and ischemic stroke. Furthermore, less frequent health monitoring and delayed medical care may exacerbate the acute impacts of air pollution (44).

Our study also found that the adverse effects of air pollutants on ischemic stroke were stronger in the cold season than in the warm season. Findings from a study conducted in Shenzhen, Guangdong Province, support our conclusion; this study reported that NO_2_ exposure during the cold season and at low temperatures was associated with a 2.75% increase in the risk of ischemic stroke hospital admissions (95% CI: 1.48–4.03%) (45). Another study performed in Chongqing used a distributed lag non-linear model (DLNM) to analyze the association between ambient air pollutants and the risk of ischemic stroke hospital admissions. It found that PM_2.5_, PM_10_, SO_2_, and NO_2_ were significantly associated with admission risk in the cold season, whereas no significant associations were observed in the warm season (22). The stronger association observed in the cold season may be attributed to poorer atmospheric diffusion conditions and higher pollutant accumulation. Meanwhile, vasoconstriction induced by low temperatures may have a synergistic interaction with air pollution, thereby increasing the risk of ischemic stroke (22).

To date, the biological mechanisms linking air pollution to ischemic stroke have not been fully elucidated, though several plausible pathways have been proposed. Air pollutants, particularly particulate matter, may elevate stroke risk by triggering systemic inflammation and oxidative stress. Such exposure can activate Toll-like receptors, provoke vascular inflammation, and further increase blood pressure (46). It may also disturb lipid metabolism and macrophage function, thereby accelerating atherosclerosis (47). Additionally, pollutant-induced pulmonary inflammation can lead to myocardial remodeling, autonomic dysfunction, and atrial fibrillation, an important precursor of ischemic stroke (48, 49). NO_2_ exposure may further exacerbate cerebral ischemic injury by regulating inflammatory mediators such as eNOS, COX-2, and ICAM-1 (50, 51). Moreover, air pollutants may impair autonomic regulation and hemodynamic stability, facilitating arrhythmia and cardiogenic embolism (52). Particulate matter can also enter the brain through the olfactory bulb, triggering neuroinflammation and eventually contributing to ischemic stroke onset (53).

Our study has several potential limitations. First, only inpatient data from Zhangzhou Municipal Hospital of Fujian Province and the Third Hospital of Zhangzhou were included, and citywide totals of ischemic stroke hospitalizations are unavailable, which prevents a precise calculation of the admission coverage rate. As the two tertiary stroke-designated centers in Zhangzhou, these two hospitals manage nearly all acute and severe ischemic stroke patients from the entire city. Although a small number of mild cases admitted to grassroots institutions were not included, such omissions are unlikely to substantially affect the temporal trend analysis or the estimated association between air pollution and ischemic stroke. Second, we used city-level ambient air pollution concentration data rather than individual-level exposure data, which may lead to exposure misclassification and thus underestimate the association between air pollution and ischemic stroke. Third, this study involved multiple statistical tests covering six air pollutants, several lag structures, and stratified subgroup analyses by gender, age and season, which may inflate the false-positive rate. Strict multiple comparison correction (e.g., Bonferroni, FDR) was not applied in the present analysis. Therefore, the relevant findings should be considered exploratory, and all significant associations need to be interpreted with caution. Fourth, other potential confounding factors—such as unhealthy lifestyles (e.g., smoking, alcohol consumption), dietary patterns, and underlying chronic diseases (e.g., hypertension, diabetes) may also influence ischemic stroke hospital admissions. However, these data were not available in our study and should be considered in future research to further refine the findings.

## Conclusions

The results indicated that short-term exposure to PM_2.5_, PM_10_, NO_2_, and O_3_ was significantly associated with daily admissions for ischaemic stroke. Males and individuals under 65 years of age were more sensitive to exposure to these air pollutants. In addition, the significant adverse effects of air pollutants were stronger in the cold season than in the warm season. During the study period, a total of 7.0% (854 cases) of ischaemic stroke admissions in Zhangzhou were attributable to air pollutant exposure exceeding the WHO standards. Our study not only provides epidemiological evidence regarding the impacts of air pollution on ischaemic stroke in subtropical cities but also offers a reference for government departments to formulate targeted air pollution intervention measures aimed at protecting vulnerable populations.

## Data Availability

The original contributions presented in the study are included in the article/Supplementary material, further inquiries can be directed to the corresponding author.
