# Supplementary material for: Attributable risk of hospital admissions for ischemic stroke due to ambient air pollution: a time-series study in Zhangzhou, China
Source: Front Public Health. 2026 Jun 17;14:1850457. doi: 10.3389/fpubh.2026.1850457 (PMC13319077; doi:10.3389/fpubh.2026.1850457)
Supplement: Supplementary file 1 [file Data_Sheet_1.doc]

Attributable risk of hospital admissions for ischemic stroke due to ambient air pollution: A time-series study in Zhangzhou, China

Yanhu Ji^1^**^*^**, Lina Zhang^1^, Yiqi Liu^1^, Yubin Chen^1^, Tongjun Chen^1^, Shengwen Wu^2^, Liang Song^3^,

**^*^** Corresponding author: Yanhu Ji; E-mail: xiaohu123596@163.com

**Supplementary materials**

**Table A1** Spearman's correlations between air pollutants and meteorological factors

**Fig.S1** Time series distributions of daily ischemic stroke admissions and environmental variables in Zhangzhou, China, 2020-2023

**Table A2** RR and 95% CI of hospital admissions for ischemic stroke with a 10 μg/m^3^ increase in concentrations of air pollutants using different lag structures in male group in Zhangzhou, China, 2020-2023

**Table A3** RR and 95% CI of hospital admissions for ischemic stroke with a 10 μg/m^3^ increase in concentrations of air pollutants using different lag structures in female group in Zhangzhou, China, 2020-2023

**Table A4** RR and 95% CI of hospital admissions for ischemic stroke with a 10 μg/m^3^ increase in concentrations of air pollutants using different lag structures in younger group (<65 years old) in Zhangzhou, China, 2020-2023

**Table A5** RR and 95% CI of hospital admissions for ischemic stroke with a 10 μg/m^3^ increase in concentrations of air pollutants using different lag structures in older group (≥65 years old ) in Zhangzhou, China, 2020-2023

**Table A6** RR and 95% CI of hospital admissions for ischemic stroke with a 10 μg/m^3^ increase in concentrations of air pollutants using different lag structures in warm season in Zhangzhou, China, 2020-2023

**Table A7** RR and 95% CI of hospital admissions for ischemic stroke with a 10 μg/m^3^ increase in concentrations of air pollutants using different lag structures in cold season in Zhangzhou, China, 2020-2023

**Table A8** Relative risks of daily ischemic stroke hospitalizations associated with per 10 μg/m^3^ increase in air pollutants in two-pollutant models

**Table A9** Sensitivity analyses for the smooth function of time trend by using different *dfs* per year

**Table A10**The results of sensitivity analyses after adjusting for other [meteorological](#C:/Users/xiaoh/AppData/Local/youdao/dict/Application/8.9.9.0/resultui/html/index.html) [factor](#C:/Users/xiaoh/AppData/Local/youdao/dict/Application/8.9.9.0/resultui/html/index.html)s (*df*=3)

**Table A1** Spearman's correlations between air pollutants and meteorological factors

|  | PM_10_ | SO_2_ | NO_2_ | CO | O_3_ | Temp | RH |
| --- | --- | --- | --- | --- | --- | --- | --- |
| PM_2.5_ | 0.9*** | 0.48*** | 0.69*** | 0.54*** | 0.42*** | -0.43*** | -0.23*** |
| PM_10_ | - | 0.57*** | 0.75*** | 0.45*** | 0.49*** | -0.38*** | -0.34*** |
| SO_2_ |  | - | 0.47*** | 0.22*** | 0.44*** | 0.017 | -0.35*** |
| NO_2_ |  |  | - | 0.46*** | 0.12*** | -0.5*** | -0.096*** |
| CO |  |  |  | - | 0.079** | -0.33*** | 0.19*** |
| O_3_ |  |  |  |  | - | 0.19*** | -0.38*** |
| Temp |  |  |  |  |  | - | 0.084** |
| RH |  |  |  |  |  |  | - |

Note. **P*<0.05, ***P*<0.01, ****P*<0.001. Temp: temperature; RH: relative humidity.

**Fig.S1** Time series distributions of daily ischemic stroke admissions and environmental variables in Zhangzhou, China, 2020–2023


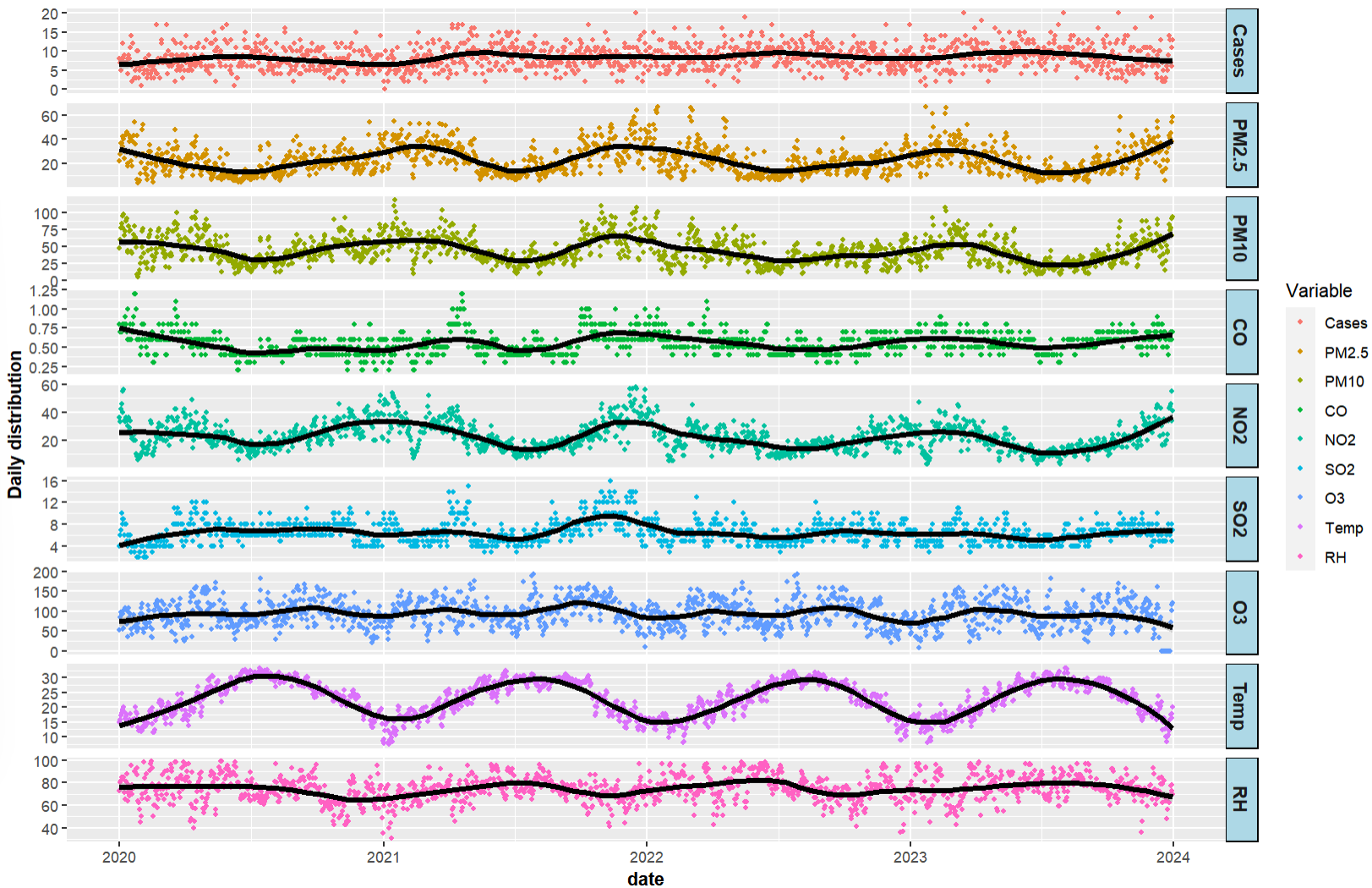
Temp: temperature; RH: relative humidity.

**Table A2** RR and 95% CI of hospital admissions for ischemic stroke with a 10 μg/m^3^ increase in concentrations of air pollutants using different lag structures in male group in Zhangzhou, China, 2020-2023

| Lag days | PM_2.5_ | PM_10_ | NO_2_ | O_3_ |
| --- | --- | --- | --- | --- |
| 0 | **1.0424 (1.0158-1.0697)^*^** | **1.0247 (1.0089-1.0408)^*^** | **1.0416 (1.0049-1.0797)^*^** | **1.0139 (1.0055-1.0224)^*^** |
| 1 | **1.0316 (1.0051-1.0587)^*^** | 1.0147 (0.9993-1.0303) | 1.0263 (0.9898-1.0641) | **1.0089 (1.0011-1.0168)^*^** |
| 2 | 1.0132 (0.9873-1.0397) | 1.0024 (0.9873-1.0175) | 1.0205 (0.9849-1.0573) | 0.9989 (0.9914-1.0066) |
| 3 | 0.9819 (0.9567-1.0077) | 0.9906 (0.9757-1.0056) | 1.0207 (0.9858-1.0569) | 0.9951 (0.9876-1.0027) |
| 4 | 0.9884 (0.9631-1.0145) | 0.9885 (0.9737-1.0035) | 0.9717 (0.9385-1.0061) | 0.9961 (0.9886-1.0036) |
| 5 | 0.9877 (0.9622-1.0138) | 0.9938 (0.9788-1.0089) | 0.9716 (0.9385-1.0059) | 0.9965 (0.9890-1.0040) |
| 01 | **1.0486 (1.0179-1.0802)^*^** | **1.0247 (1.0071-1.0426)^*^** | **1.0443 (1.0024-1.0881)^*^** | **1.0147 (1.0054-1.0241)^*^** |
| 02 | **1.0471 (1.0130-1.0823)^*^** | **1.0206 (1.0014-1.0401)^*^** | **1.0458 (1.0002-1.0934)^*^** | **1.0107 (1.0007-1.0208)^*^** |
| 03 | 1.0322 (0.9954-1.0703) | 1.0131 (0.9926-1.0340) | 1.0481 (0.9998-1.0987) | 1.0067 (0.9960-1.0174) |
| 04 | 1.0246 (0.9852-1.0656) | 1.0071 (0.9853-1.0293) | 1.0300 (0.9803-1.0823) | 1.0042 (0.9929-1.0156) |
| 05 | 1.0194 (0.9773-1.0633) | 1.0046 (0.9816-1.0281) | 1.0152 (0.9643-1.0689) | 1.0026 (0.9907-1.0146) |

Note: ^*^*P*<0.05

**Table A3** RR and 95% CI of hospital admissions for ischemic stroke with a 10 μg/m^3^ increase in concentrations of air pollutants using different lag structures in female group in Zhangzhou, China, 2020-2023

| Lag days | PM_2.5_ | PM_10_ | NO_2_ | O_3_ |
| --- | --- | --- | --- | --- |
| 0 | 1.0112 (0.9766-1.0469) | 1.0118 (0.9909-1.0331) | 1.0265 (0.9791-1.0762) | 1.0067 (0.9954-1.0181) |
| 1 | 1.0092 (0.9747-1.0451) | 1.0052 (0.9850-1.0258) | 1.0206 (0.9730-1.0704) | 0.9986 (0.9881-1.0092) |
| 2 | 0.9884 (0.9548-1.0232) | 0.9984 (0.9785-1.0185) | 1.0008 (0.9549-1.0490) | 1.0002 (0.9899-1.0104) |
| 3 | 0.9886 (0.9551-1.0232) | 1.0033 (0.9835-1.0235) | 0.9812 (0.9369-1.0274) | 1.0055 (0.9953-1.0157) |
| 4 | 0.9958 (0.9620-1.0308) | 1.0024 (0.9825-1.0225) | 0.9824 (0.9383-1.0285) | 1.0003 (0.9903-1.0105) |
| 5 | 0.9742 (0.9408-1.0088) | 0.9918 (0.9721-1.0119) | 0.9886 (0.9441-1.0352) | 0.9946 (0.9846-1.0047) |
| 01 | 1.0140 (0.9743-1.0553) | 1.0111 (0.9879-1.0347) | 1.0303 (0.9759-1.0876) | 1.0032 (0.9907-1.0159) |
| 02 | 1.0055 (0.9618-1.0512) | 1.0081 (0.9830-1.0339) | 1.0244 (0.9657-1.0868) | 1.0026 (0.9892-1.0162) |
| 03 | 0.9988 (0.9515-1.0486) | 1.0084 (0.9814-1.0361) | 1.0116 (0.9502-1.0769) | 1.0050 (0.9907-1.0195) |
| 04 | 0.9971 (0.9461-1.0507) | 1.0087 (0.9799-1.0382) | 1.0029 (0.9392-1.0708) | 1.0046 (0.9895-1.0200) |
| 05 | 0.9861 (0.9321-1.0432) | 1.0050 (0.9746-1.0363) | 0.9981 (0.9321-1.0686) | 1.0021 (0.9861-1.0182) |

Note: ^*^*P*<0.05

**Table A4** RR and 95% CI of hospital admissions for ischemic stroke with a 10 μg/m^3^ increase in concentrations of air pollutants using different lag structures in younger group (<65 years old) in Zhangzhou, China, 2020-2023

| Lag days | PM_2.5_ | PM_10_ | NO_2_ | O_3_ |
| --- | --- | --- | --- | --- |
| 0 | **1.0371 (1.0037-1.0717)**^*^ | **1.0286 (1.0086-1.0491)**^*^ | 1.0363 (0.9902-1.0845) | **1.0166 (1.0059-1.0274)**^*^ |
| 1 | 1.0243 (0.9911-1.0587) | 1.0108 (0.9915-1.0306) | 1.0044 (0.9594-1.0515) | 1.0080 (0.9981-1.0180) |
| 2 | 1.0001 (0.9678-1.0334) | 0.9947 (0.9758-1.0138) | 1.0065 (0.9622-1.0528) | 0.9948 (0.9853-1.0045) |
| 3 | 0.9864 (0.9545-1.0192) | 0.9988 (0.9800-1.0179) | 1.0058 (0.9624-1.0511) | 0.9967 (0.9872-1.0063) |
| 4 | 1.0101 (0.9776-1.0437) | 1.0020 (0.9831-1.0213) | 0.9979 (0.9551-1.0427) | 0.9995 (0.9901-1.0091) |
| 5 | 0.9973 (0.9649-1.0307) | 0.9980 (0.9792-1.0173) | 0.9912 (0.9486-1.0357) | 0.9964 (0.9870-1.0059) |
| 01 | **1.0413 (1.0029-1.0812)**^*^ | **1.0253 (1.0031-1.0481)**^*^ | 1.0258 (0.9739-1.0806) | **1.0161 (1.0042-1.0281)**^*^ |
| 02 | 1.0332 (0.9908-1.0775) | 1.0166 (0.9926-1.0413) | 1.0236 (0.9674-1.0831) | 1.0093 (0.9966-1.0221) |
| 03 | 1.0226 (0.9767-1.0706) | 1.0138 (0.9879-1.0403) | 1.0224 (0.9632-1.0854) | 1.0062 (0.9927-1.0199) |
| 04 | 1.0258 (0.9762-1.0779) | 1.0134 (0.9858-1.0417) | 1.0188 (0.9570-1.0846) | 1.0054 (0.9911-1.0199) |
| 05 | 1.0251 (0.9719-1.0811) | 1.0123 (0.9831-1.0423) | 1.0124 (0.9485-1.0805) | 1.0036 (0.9886-1.0189) |

Note: ^*^*P*<0.05

**Table A5** RR and 95% CI of hospital admissions for ischemic stroke with a 10 μg/m^3^ increase in concentrations of air pollutants using different lag structures in older group (≥65 years old ) in Zhangzhou, China, 2020-2023

| Lag days | PM_2.5_ | PM_10_ | NO_2_ | O_3_ |
| --- | --- | --- | --- | --- |
| 0 | 1.0264 (0.9992-1.0544) | 1.0144 (0.9981-1.0309) | 1.0354 (0.9980-1.0742) | 1.0081 (0.9993-1.0168) |
| 1 | 1.0223 (0.9951-1.0502) | 1.0113 (0.9955-1.0273) | 1.0358 (0.9982-1.0749) | 1.0035 (0.9953-1.0117) |
| 2 | 1.0064 (0.9798-1.0336) | 1.0047 (0.9892-1.0205) | 1.0168 (0.9803-1.0545) | 1.0023 (0.9944-1.0103) |
| 3 | 0.9830 (0.9571-1.0097) | 0.9930 (0.9777-1.0085) | 1.0057 (0.9703-1.0423) | 1.0003 (0.9925-1.0082) |
| 4 | 0.9792 (0.9533-1.0059) | 0.9883 (0.9731-1.0038) | 0.9616 (0.9279-1.0001) | 0.9964 (0.9887-1.0042) |
| 5 | 0.9738 (0.9478-1.0004) | 0.9900 (0.9747-1.0056) | 0.9693 (0.9352-1.0046) | 0.9955 (0.9878-1.0033) |
| 01 | 1.0318 (0.9999-1.0640) | 1.0160 (0.9979-1.0344) | 1.0466 (0.9998-1.0916) | 1.0072 (0.9976-1.0170) |
| 02 | 1.0301 (0.9954-1.0661) | 1.0154 (0.9957-1.0354) | 1.0459 (0.9991-1.0949) | 1.0069 (0.9966-1.0174) |
| 03 | 1.0176 (0.9801-1.0564) | 1.0097 (0.9887-1.0311) | 1.0411 (0.9918-1.0928) | 1.0061 (0.9951-1.0172) |
| 04 | 1.0068 (0.9667-1.0484) | 1.0040 (0.9817-1.0267) | 1.0196 (0.9691-1.0728) | 1.0038 (0.9922-1.0157) |
| 05 | 0.9954 (0.9529-1.0397) | 0.9999 (0.9764-1.0241) | 1.0057 (0.9538-1.0604) | 1.0018 (0.9895-1.0143) |

Note: ^*^*P*<0.05

Table A6 RR and 95% CI of hospital admissions for ischemic stroke with a 10 μg/m3 increase in concentrations of air pollutants using different lag structures in warm season in Zhangzhou, China, 2020-2023

| Lag days | PM_2.5_ | PM_10_ | NO_2_ | O_3_ |
| --- | --- | --- | --- | --- |
| 0 | 1.0048 (0.9598-1.0519) | 1.0221 (0.9945-1.0506) | 1.0514 (0.9778-1.1305) | 1.0063 (0.9952-1.0175) |
| 1 | 0.9962 (0.9519-1.0424) | 1.0111 (0.9842-1.0387) | **1.0765 (1.0041-1.1541)^*^** | 0.9965 (0.9858-1.0072) |
| 2 | 0.9662 (0.9227-1.0117) | 0.9896 (0.9632-1.0168) | 1.0084 (0.9406-1.0810) | 0.9958 (0.9852-1.0065) |
| 3 | 0.9578 (0.9147-1.0028) | 0.9922 (0.9659-1.0193) | 1.0063 (0.9397-1.0775) | 0.9954 (0.9848-1.0061) |
| 4 | 0.9750 (0.9314-1.0206) | 0.9892 (0.9628-1.0163) | 0.9500 (0.8868-1.0177) | 0.9979 (0.9875-1.0085) |
| 5 | 0.9905 (0.9461-1.0369) | 1.0037 (0.9768-1.0313) | 0.9919 (0.9251-1.0635) | 0.9995 (0.9891-1.0101) |
| 01 | 1.0006 (0.9499-1.0539) | 1.0213 (0.9903-1.0534) | **1.0883 (1.0019-1.1821)^*^** | 1.0015 (0.9892-1.0141) |
| 02 | 0.9823 (0.9267-1.0411) | 1.0118 (0.9776-1.0472) | 1.0763 (0.9822-1.1795) | 0.9990 (0.9855-1.0127) |
| 03 | 0.9638 (0.9043-1.0272) | 1.0068 (0.9695-1.0454) | 1.0697 (0.9690-1.1808) | 0.9969 (0.9823-1.0118) |
| 04 | 0.9554 (0.8920-1.0232) | 1.0014 (0.9613-1.0430) | 1.0380 (0.9339-1.1537) | 0.9962 (0.9805-1.0122) |
| 05 | 0.9533 (0.8856-1.0261) | 1.0030 (0.9597-1.0481) | 1.0324 (0.9222-1.1559) | 0.9962 (0.9794-1.0133) |

Note: ^*^*P*<0.05

**Table A7** RR and 95% CI of hospital admissions for ischemic stroke with a 10 μg/m^3^ increase in concentrations of air pollutants using different lag structures in cold season in Zhangzhou, China, 2020-2023

| Lag days | PM_2.5_ | PM_10_ | NO_2_ | O_3_ |
| --- | --- | --- | --- | --- |
| 0 | **1.0377 (1.0058-1.0706)^*^** | **1.0275 (1.0079-1.0475)^*^** | **1.0479 (1.0037-1.0942)^*^** | **1.0202 (1.0063-1.0341)^*^** |
| 1 | **1.0321 (1.0001-1.0652)^*^** | **1.0194 (1.0003-1.0389)^*^** | 1.0175 (0.9729-1.0642) | **1.0154 (1.0033-1.0277)^*^** |
| 2 | 1.0171 (0.9859-1.0492) | 1.0129 (0.9943-1.0319) | 1.0253 (0.9812-1.0715) | 1.0014 (0.9899-1.0129) |
| 3 | 0.9933 (0.9627-1.0247) | 1.0024 (0.9839-1.0211) | 1.0128 (0.9703-1.0571) | 1.0012 (0.9899-1.0127) |
| 4 | 0.9976 (0.9668-1.0292) | 0.9991 (0.9808-1.0178) | 0.9843 (0.9433-1.0271) | 0.9938 (0.9829-1.0048) |
| 5 | 0.9805 (0.9502-1.0118) | 0.9907 (0.9725-1.0092) | 0.9740 (0.9337-1.0161) | 0.9871 (0.9764-1.0001) |
| 01 | **1.0473 (1.0099-1.0860)^*^** | **1.0303 (1.0083-1.0528)^*^** | 1.0447 (0.9927-1.0995) | **1.0251 (1.0096-1.0407)^*^** |
| 02 | **1.0497 (1.0077-1.0934)^*^** | **1.0314 (1.0072-1.0562)^*^** | 1.0511 (0.9931-1.1126) | **1.0199 (1.0035-1.0366)^*^** |
| 03 | 1.0406 (0.9947-1.0886) | **1.0283 (1.0022-1.0552)^*^** | 1.0502 (0.9881-1.1162) | **1.0177 (1.0003-1.0356)^*^** |
| 04 | 1.0377 (0.9876-1.0903) | 1.0258 (0.9977-1.0548) | 1.0375 (0.9724-1.1068) | 1.0124 (0.9940-1.0313) |
| 05 | 1.0289 (0.9752-1.0856) | 1.0211 (0.9912-1.0517) | 1.0226 (0.9555-1.0944) | 1.0047 (0.9854-1.0242) |

Note: ^*^*P*<0.05

**Table A8** Relative risks of daily ischemic stroke hospitalizations associated with per 10 μg/m^3^ increase in air pollutants in two-pollutant models

| Model | RR | 95% CI |
| --- | --- | --- |
| PM_2.5_ |  |  |
| Single pollutant model | 1.0356 | 1.0107-1.0611* |
| +SO_2_ | 1.0358 | 1.0089-1.0634* |
| +NO_2_ | 1.0295 | 1.0011-1.0586* |
| +CO | 1.0354 | 1.0069-1.0647* |
| +O_3_ | 1.0228 | 0.9958-1.0505 |
| PM_10_ |  |  |
| Single pollutant model | 1.0198 | 1.0069-1.0329* |
| +SO_2_ | 1.0248 | 1.0093-1.0406* |
| +NO_2_ | 1.0198 | 1.0023-1.0377* |
| +CO | 1.0187 | 1.0043-1.0333* |
| +O_3_ | 1.0121 | 0.9970-1.0274 |
| NO_2_ |  |  |
| Single pollutant model | 1.0387 | 1.0045-1.0740* |
| +PM_2.5_ | 1.0162 | 0.9770-1.0570 |
| +PM_10_ | 1.0107 | 0.9693-1.0539 |
| +SO_2_ | 1.0396 | 1.0004-1.0805* |
| +CO | 1.0338 | 0.9972-1.0719 |
| +O_3_ | 1.0306 | 0.9962-1.0662 |
| O_3_ |  |  |
| Single pollutant model | 1.0114 | 1.0044-1.0184* |
| +PM_2.5_ | 1.0078 | 1.0001-1.0161* |
| +PM_10_ | 1.0088 | 1.0004-1.0172* |
| +SO_2_ | 1.0125 | 1.0052-1.0199* |
| +CO | 1.0111 | 1.0038-1.0185* |
| +NO_2_ | 1.0122 | 1.0049-1.0195* |

**Note:** **P* < 0.05.

The strongest effects of air pollutants in single-pollutant models were used (PM_2.5_, lag01 day; PM_10_, lag0 day; SO_2_, lag7 day; NO_2_, lag01 day; O_3_, lag0 day)

**Table A9** Sensitivity analyses for the smooth function of time trend by using different *dfs* per year

| *df* | PM_2.5_ | PM_10_ | NO_2_ | O_3_ |
| --- | --- | --- | --- | --- |
| 5 | 1.0291 (1.0053-1.0534)* | 1.0171 (1.0047-1.0296)* | 1.0309 (1.0001-1.0633)* | 1.0101 (1.0038-1.0174)* |
| 6 | 1.0317 (1.0073-1.0565)* | 1.0193 (1.0065-1.0323)* | 1.0371 (1.0040-1.0713)* | 1.0112 (1.0043-1.0182)* |
| 7 | 1.0356 (1.0107-1.0611)* | 1.0198 (1.0069-1.0329)* | 1.0387(1.0045-1.0740)* | 1.0114 (1.0044-1.0184)* |
| 8 | 1.0370 (1.0119-1.0626)* | 1.0190 (1.0061-1.0321)* | 1.0361 (1.0008-1.0725)* | 1.0118 (1.0048-1.0188)* |
| 9 | 1.0368 (1.0115-1.0628)* | 1.0193 (1.0063-1.0324)* | 1.0367 (1.0001-1.0746)* | 1.0119 (1.0049-1.0189)* |

**Note:** **P* < 0.05. The strongest effects of air pollutants in single-pollutant models were used (PM_2.5_, lag01 day; PM_10_, lag0 day; NO_2_, lag01 day; O_3_, lag0 day)

**Table A10** The results of sensitivity analyses after adjusting for other [meteorological](#C:/Users/xiaoh/AppData/Local/youdao/dict/Application/8.9.9.0/resultui/html/index.html) [factor](#C:/Users/xiaoh/AppData/Local/youdao/dict/Application/8.9.9.0/resultui/html/index.html)s (*df*=3)

| **Factors** | Model used in the article^＃^ | + [Precipitation](#C:/Users/xiaoh/AppData/Local/youdao/dict/Application/8.9.9.0/resultui/html/index.html) | + Sunshine duration | + Wind speed |
| --- | --- | --- | --- | --- |
| PM_2.5_ | 1.0356 (1.0107-1.0611)* | 1.0347 (1.0097-1.0602)* | 1.0347 (1.0097-1.0603)* | 1.0361 (1.0108-1.0619)* |
| PM_10_ | 1.0198 (1.0069-1.0329)* | 1.0187 (1.0058-1.0319)* | 1.0190 (1.0060-1.0322)* | 1.0206 (1.0074-1.0340)* |
| NO_2_ | 1.0387(1.0045-1.0740)* | 1.0356 (1.0014-1.0701)* | 1.0361 (1.0016-1.0717)* | 1.0378 (1.0033-1.0736)* |
| O_3_ | 1.0114 (1.0044-1.0184)* | 1.0113 (1.0043-1.0183)* | 1.0110 (1.0037-1.0184)* | 1.0125 (1.0055-1.0196)* |

^＃^The model covariates include long-term and seasonal trends, average temperature, relative humidity, day of the week and holiday effects.

**P*<0.05; PM_2.5_, lag01 day; PM_10_, lag0 day; NO_2_, lag01 day; O_3_, lag0 day
